# The Impact of a Workplace Terrorist Attack on the Psychosocial Work Environment: A Longitudinal Study From Pre- to Post-disaster

**DOI:** 10.3389/fpubh.2021.708260

**Published:** 2021-11-04

**Authors:** Mona Berthelsen, Marianne Bang Hansen, Alexander Nissen, Morten Birkeland Nielsen, Stein Knardahl, Trond Heir

**Affiliations:** ^1^Norwegian Centre for Violence and Traumatic Stress Studies, Oslo, Norway; ^2^Norwegian National Unit for Hearing Impairment and Mental Health, Oslo University Hospital, Oslo, Norway; ^3^Department of Work Psychology and Physiology, National Institute of Occupational Health, Oslo, Norway; ^4^Institute of Clinical Medicine, Faculty of Medicine, University of Oslo, Oslo, Norway

**Keywords:** terrorism, disaster, workplace, work environment, role clarity, role conflict, predictability, organizational resilience

## Abstract

The psychosocial work environment is of great importance for regaining health and productivity after a workplace disaster. Still, there is a lack of knowledge about the impact of a disaster on the psychosocial work environment. The purpose of this study was to examine whether employees' perceptions of role clarity, role conflicts, and predictability in their work situation changed from before to after a workplace terrorist attack. We combined data from two prospective work environment surveys of employees in three governmental ministries that were the target of the 2011 Oslo terrorist attack. A first two-wave survey was conducted 4–5 years and 2–3 years before the attack, and a second three-wave survey took place 10 months, 2 years, and 3 years after the attack. Of 504 individuals who were employed at the time of the bombing, 220 were employed in both pre- and post-disaster periods, participated in both the first and the second survey, and consented to the linking of data from the two surveys. We found no significant changes in levels of role clarity, role conflict, and predictability from before to after the terrorist attack. Adjusting for sex, age and education had no effect on the results. The findings suggest that perceptions of the psychosocial working environment are likely to be maintained at previous levels in the aftermath of a workplace disaster. Considering the importance of the psychosocial work environment for regaining health and productivity, the findings are important for the preparation for, and management of, future crises.

## Introduction

Over the last two decades, workplaces around the world have been increasingly exposed to natural and man-made disasters that destroy businesses, disrupt productivity and result in economic, social and health consequences (1). To maintain health and productivity in the aftermath of such events, the psychosocial work environment is important. Following the 2011 Oslo terrorist attack directed toward the governmental ministries in Norway, employees' appraisals of lower role conflicts, higher role clarity, higher predictability, and higher leader support were associated with lower psychological distress (2). Control over decisions at work and support from superiors and co-workers reduced the odds of sick leave, whereas experiences of role conflicts increased the odds (3). Even for those who met the symptom criteria for post-traumatic stress disorder (PTSD), predictability and control over decisions at work reduced the probability of sick leave (4).

A disaster can have devastating effects on a workplace, resulting in a need for reconstruction, relocation, and restructuring of the work organization. Psychological stress reactions following the disaster may have the potential to affect the efficiency and interactions in the organization for a long time (1, 5). Despite the importance of the work environment for health and productivity, there is a lack of knowledge about the impact of a workplace disaster on the psychosocial work environment.

We have previously reported that employees' perceptions of leadership and supportive management were remarkably stable from before to after the 2011 Oslo bombing (6). For multinational employees who were not directly affected by the September 11 attacks in the US, little evidence was fond for consequences of the terror attack on perceptions of their jobs and organizations (7). To the best of our knowledge, there are no other studies that have examined psychosocial work environment factors before and after a workplace disaster, natural or man-made. It seems relevant to examine changes in role clarity, role conflicts, and predictability because of their importance for workers' health and work participation in the aftermath of a terrorist attack (2–4); especially if this can be done in an organization that has been directly affected by disaster.

Role clarity refers to having clearly defined expectations and objectives of tasks and responsibilities at work; role conflict is defined as the experience of conflicting demands and insufficient resources e.g., when a person has several conflicting roles or receives conflicting messages from others; and predictability refers to a worker's ability to anticipate challenges and future demands (8).

The context of the present study was the Norwegian government ministries that were hit by a terrorist attack on July 22, 2011. A bomb blast shattered the government buildings, killing eight people and injuring 209 others. Based on data from work environment surveys carried out prior to and after the incident, the purpose of the study was to examine whether employees' perceptions of role clarity, role conflicts, and predictability in their work situation changed from before to after the terrorist attack.

## Methods

### Design and Participants

In the present study we have combined data from two prospective surveys of employees in three governmental ministries who participated in research on their working environment both before and after the bombing. The first survey took place before the terrorist attack and consisted of two waves of data collection: Pre-1 (4–5 years prior to the event) and Pre-2 (2–3 years prior to the event) (9). The second survey took place after the terrorist attack and consisted of three waves of data collection: Post-1 (10 months post event), Post-2 (2 years post event), and Post-3 (3 years post event) (10). In both surveys, employees were asked to respond voluntarily to a web-based questionnaire for examinations of the work environment as part of routine follow-up of health, environment, and safety in their organization (9, 10).

**Figure 1:**
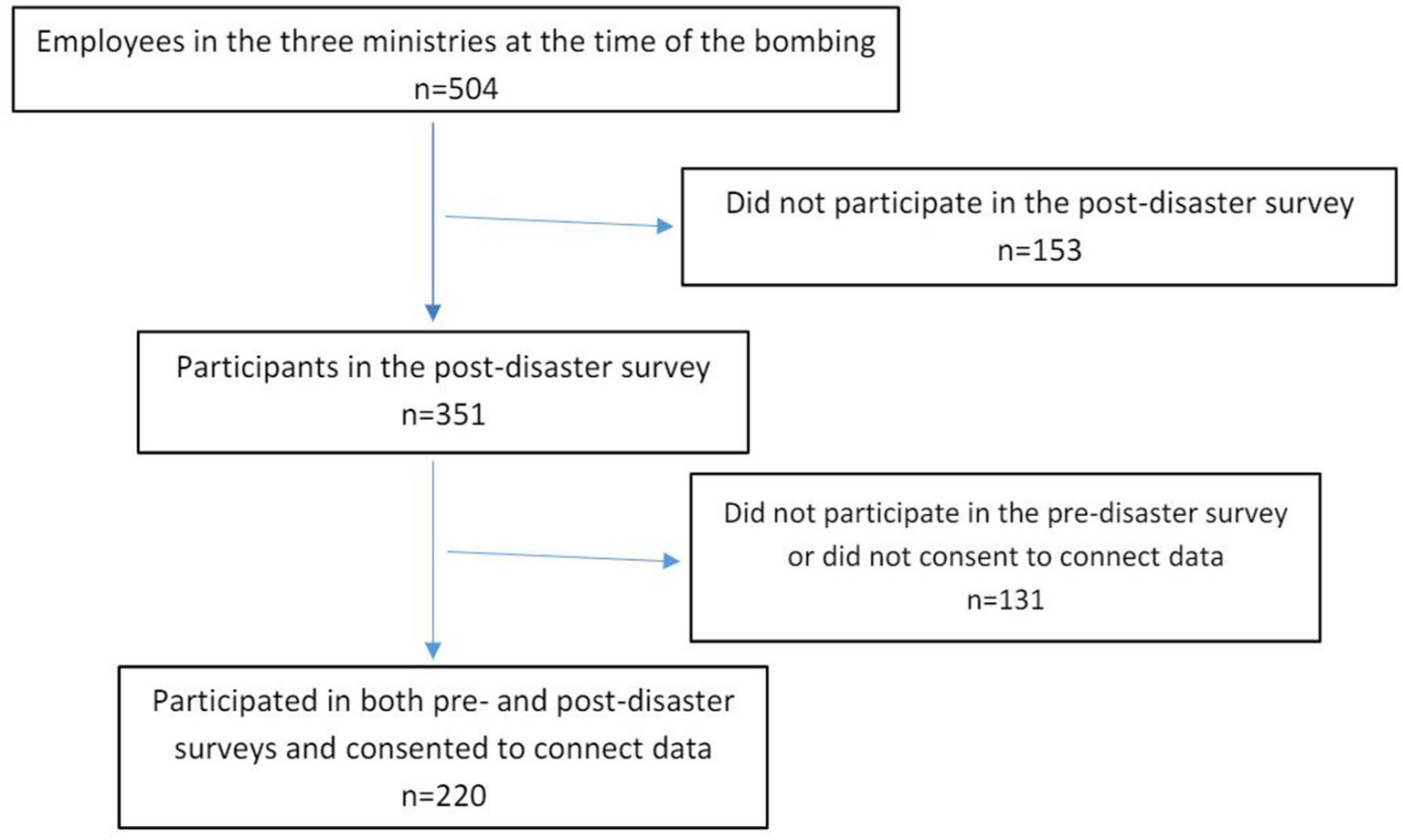
Flowchart showing the inclusion of participants.

Of the 504 individuals employed in the three ministries at the time of the bombing, 220 met the following three criteria and were included in the study: They were employed by the ministry in both pre- and post-disaster periods, they participated in both pre- and post-disaster surveys, and they consented to the linking of data from the two survey periods (Figure 1). Unfortunately, we do not know how many of the 504 individuals employed at the time of the attack who were also employed in both the pre and post time periods. Therefore, we are unable to estimate an exact response rate.

### Measures

In both surveys, data were collected from a web-based questionnaire. Information was obtained on sex, age, education, exposure to the bomb blast, and experiences with the psychosocial work environment (9, 10).

Role clarity, role conflict, and predictability were measured by the General Questionnaire for Psychological and Social factors at Work, QPSNordic (8). Each of the three factors comprised three items. Examples of the items were “Do you know exactly what is expected of you at work?” (role clarity), “Do you receive incompatible requests from two or more people?” (role conflict), and “Do you know in advance what kind of tasks to expect a month from now?” (predictability). The response categories for all items referred to the frequency of occurrence: 1 (Very seldom or never), 2 (Rather seldom), 3 (Sometimes), 4 (Rather often) and 5 (Very often or always). The internal consistencies of the scales were satisfactory (Cronbach's alphas Pre-1 to Post-3, role clarity: 0.79, 0.77, 0.80, 0.80, and 0.78; role conflict: 0.63, 0.62, 0.65, 0.58, and 0.60; and predictability: 0.58, 0.39, 0.54, 0.50, and 0.61). We calculated the average score for each factor. A maximum of one missing response on each factor was allowed, which yielded 220 valid questionnaires.

### Procedures

Prior to the data collections, employees and managers were informed about the survey aims and data collection procedures. Subsequently, all employees were mailed a letter with information about the survey as well as a personal log-in code to the web-questionnaire. The written information explained the aims of the study, confidentiality issues and consent procedures, all in accordance with relevant guidelines and approved by the Norwegian Data Inspectorate. Informed consent was given by the respondents. Employees had the opportunity to fill out the questionnaire at work, from home or anywhere with internet access. Respondents were able to log on to the web questionnaire an unlimited number of times to change or to complete the survey during data collection. The surveys were approved by the Regional Committee for Medical and Health Research Ethics, Southeast, Norway (reference number: 2011/1577).

### Organizational Context

Due to extensive damage to buildings and facilities, ministries A (*n* = 101) and B (*n* = 82) had to relocate immediately after the bomb explosion. Ministry B relocated three more times in the period up to Post-3. Ministry A changed its office structure from cell offices to open-plan offices between Post-1 and Post-2, and back to cell offices between Post-2 and Post-3. Ministry C (*n* = 37) suffered limited damage from the explosion and stayed in the same building throughout the post-explosion data collection period.

Between Post-2 and Post-3, a new national government was elected. This may have resulted in reorganization of some work tasks. However, ministry employees in Norway continue in their positions as bureaucrats regardless of whether a new political government is elected.

### Statistical Analyses

We estimated changes in role clarity, role conflict, and predictability across time of assessment using Linear Mixed Effects analyses in R with the package nlme: Linear and Non-linear Mixed Effects Models version 3.1-131 (11). Role clarity, role conflict, and predictability were entered as fixed effects in three separate models. We modeled random intercepts for subjects. The five times of assessment were modeled as a categorical variable and did not allow the calculation of random slopes.

## Results

There were slightly more female responders (*n* = 124, 56%) than males (*n* = 96, 44%). Average age was 46.9 years at the time of the terror attack (*SD* = 10.3, range 29–69). Most of the participants had more than 16 years of education (*n* = 162, 74%), a minority had 13 to 16 years of education (*n* = 43, 20%) or less (*n* = 15, 7%).

All assessments of role clarity and predictability were skewed toward higher levels than the theoretical mean of 3.0, whereas assessments of role conflict were skewed toward lower levels (Table 1).

**Table 1:** Descriptive statistics of role clarity, role conflict and predictability in n=220 ministerial employees before and after the 2011 Oslo bombing.

	**Pre-1**			**Pre-2**			**Post-1**			**Post-2**			**Post-3**		
	**Mean**	**SD**	**Min-max**	**Mean**	**SD**	**Min-max**	**Mean**	**SD**	**Min-max**	**Mean**	**SD**	**Min-max**	**Mean**	**SD**	**Min-max**
**Role clarity**	3.83	0.79	1.33–5.00	3.83	0.72	1.00–5.00	3.80	0.73	1.67–5.00	3.85	0.75	1.00–5.00	4.05	0.65	2.33–5.00
**Role conflict**	2.45	0.70	1.00–4.00	2.34	0.66	1.00–4.33	2.43	0.68	1.00–4.00	2.33	0.67	1.00–4.00	2.29	0.65	1.00–4.00
**Predictability**	4.18	0.60	1.00–5.00	4.22	0.52	2.67–5.00	4.21	0.53	1.67–5.00	4.28	0.52	2.67–5.00	4.32	0.54	1.00–5.00

*Pre-1 (4–5 years prior to event); Pre-2 (2–3 years prior to event); Post-1 (10 months post event); Post-2 (2 years post event); Post-3 (3 years post event)*.

There were no statistically significant changes in levels of role clarity, role conflict, and predictability from Pre-2 to any post-disaster assessment (Table 2). Adjustment for gender, age and education did not change these results.

**Table 2:** Estimates^a^ of change in levels of role clarity, role conflict and predictability in *n* = 220 ministerial employees before and after the 2011 Oslo bombing.

	**Fixed effects**				
	**Coefficient**	**95% CI**	**SE**	***t*** **-value**	***p*** **-value**
**Role clarity:**					
Pre-1^b^	−0.03	−0.15–0.09	0.061	−0.51	0.613
Pre-2	Ref				
Post-1	−0.08	−0.20–0.03	0.058	−1.43	0.151
Post-2	0.00	−0.12–0.12	0.059	0.00	0.994
Post-3	0.11	−0.01–0.23	0.061	1.857	0.064
**Role conflict:**					
Pre-1	0.10	−0.01–0.22	0.058	1.75	0.081
Pre-2	Ref				
Post-1	0.06	−0.05–0.17	0.056	1.14	0.256
Post-2	−0.03	−0.14–0.08	0.057	−0.55	0.581
Post-3	−0.00	−0.12–0.11	0.059	−0.05	0.957
**Predictability:**					
Pre-1	−0.08	−0.18–0.01	0.048	−1.75	0.081
Pre-2	Ref				
Post-1	−0.05	−0.14–0.05	0.046	−0.98	0.328
Post-2	0.02	−0.06–0.12	0.046	0.60	0.548
Post-3	0.06	−0.03–0.16	0.048	1.28	0.201

a *Linear mixed effects analyses*.

b *Pre-1 (4–5 years prior to event); Pre-2 (2–3 years prior to event); Post-1 (10 months post event); Post-2 (2 years post event); Post-3 (3 years post event)*.

*Intercepts, y [95% CI]: Role clarity 3.87 [3.77–3.98], Role conflict 2.34 [2.24–2.44], Predictability 4.24 [4.16–4.32]*.

*Random effects, SE [95% CI]: Role clarity 0.51 [0.48–0.54], Role conflict 0.49 [0.46–0.52], Predictability 0.40 [0.38–0.43]*.

## Discussion

We examined the impact of a workplace terrorist attack on the psychosocial work environment in terms of employees' perception of role clarity, role conflicts, and work predictability before and after the attack. Overall, appraisal of role expectations and predictability appeared to be unchanged from before to after the attack.

In general, affective reactions can have a major impact on an organization as they influence attitudes and behavior directly (12). In the long run, emotions can manifest themselves in job satisfaction or work productivity (13). Thus, several factors could have influenced employees' perception of predictability and role expectations, such as relocations, loss of infrastructure and personal property (14), increase in sick leave (15), acquired post-traumatic stress reactions (10), concerns about safety at work (16), experiences of high work demand (17), and disagreement in the workforce about who deserved relief and support the most (17).

Nevertheless, and despite the requirement for rapid problem solving, flexibility and restructuring, our findings show that it is possible to maintain previous levels of perceived predictability and role expectations after a workplace terrorist attack. The findings are complementary to the study of employees' perceptions of leadership and supportive management which were also remarkably stable from before to after the attack (6) and indicate that the psychosocial work environment can be inherently stable and resistant to changes caused by external causes.

At the organizational level, it is interesting to evaluate the stability in perceptions of the working environment in the light of the concept of organizational resilience, most often understood as the organization's ability to deal with internal and external changes, risks or jolts (18). Stability in perceptions may reflect that the three ministries in focus were well-functioning resilient organizations that were able to emerge from challenging conditions (19), continue to meet their objectives in the face of challenges (20), or bounce back after a catastrophic event (21).

The literature is still far from reaching consensus on what makes an organization resilient (22). Among the most cited characteristics are material resources, human capital, preparedness and planning, information management, governance processes, leadership practices, organizational culture, social networks, flexibility, and collaboration (18, 20). Norway is a high-income, politically stable country with mature democracy and easy access to material resources. The ministry employees were, on average, highly educated with associated expectations of human capital, flexibility, and collaboration skills. Thus, some basic conditions may have been in favor of good organizational resilience in the ministries in focus. Future research should examine the stability of the working environment through crises in other countries and under different socio-economic conditions.

Alternatively, employees' perceptions of the psychosocial work environment may be rooted in cognitive schemas that are more resistant to change than the work environment itself. As suggested by Ryan et al. (7), even strong stress reactions or emotions related to a terrorist attack do not need to shake the more stable appraisals about one's job. The exceptional nature of a terrorist attack may also have resulted in the employees adapting to a different template post disaster, against which to compare their work environment (7).

### Strengths and Limitations

Strengths of the study include the use of a longitudinal design comparing data from before and after the terrorist attack, and the use of a well-validated instrument (8) to measure employees' experience of role clarity, role conflict, and predictability.

Study limitations include a relatively small study sample size. Thus, the power was too low for multilevel models that could consider different working groups, departments, or ministries. Second, our sample consisted mainly of highly educated ministry employees in a high income, political stable country, which may question the generalizability of our findings. Third, we were not able to calculate the response rate since we do not know how many of those who worked in the ministry in the post disaster period who had also been employed during the previous waves of data collection. This may have led to less insight into issues with selection bias.

Fourth, other changes or processes may have occurred during the study period. For example, two interventions were carried out to alleviate long-term consequences of the terrorist attack. The ministerial leaders were offered a training program on how to recognize and acknowledge stress reactions among their subordinates (1), and the occupational health service screened those most heavily exposed to the bomb blast for physical and psychological symptoms (23). Unfortunately, we have no evidence about the effects of these interventions.

We also do not know the effect of the continuous feedback given to the ministries about findings from the surveys. Finally, we cannot distinguish whether stability in self-reported data was related to behavioral or perceptual mechanisms.

### Implications

Perceptions of the psychosocial work environment is important for maintaining health and productivity after a workplace disaster (2–4, 17). Our findings that previous levels of perceived predictability and role expectations were maintained after a terrorist attack are in that sense a positive message with important implications. The foundation for success in future crises may be in the current organization, in that the perceptions of a well-functioning work environment can be continued. Thus, arguments for improving the psychosocial work environment in times of normality should be supported in disaster planning.

## Conclusion

Overall, appraisals of role expectations and predictability were remarkably stable from before to after the 2011 Oslo terrorist attack. This suggests that it is possible to maintain perceptions of the psychosocial working environment at previous levels in the aftermath of a workplace disaster. The knowledge is important for the planning and management of future crises.

## Data Availability Statement

Data are not publicly available due to confidentiality agreements with participants. However, data is available to interested researchers upon request, pending ethical approval from the Norwegian Regional committees for medical and health research ethics. Data is to be stored properly and in line with the Norwegian Law of privacy protection. Requests to access the datasets should be directed to trond.heir@medisin.uio.no.

## Ethics Statement

The studies involving human participants were reviewed and approved by the Regional Committee for Medical and Health Research Ethics, South East, Norway (reference number: 2011/1577). The patients/participants provided their written informed consent to participate in this study.

## Author Contributions

TH, SK, MH, and MN designed the study. MB and AN prepared the dataset and analyzed the data. MB and TH drafted the paper. All authors contributed to the revision of the manuscript and approved the final version.

## Funding

The current study was funded by the Research council of Norway, grant number 227 039. The funders had no role in design of the study, data collection, analysis and interpretation of the data, or in the decision to submit the paper for publication.

## Conflict of Interest

The authors declare that the research was conducted in the absence of any commercial or financial relationships that could be construed as a potential conflict of interest.

## Publisher's Note

All claims expressed in this article are solely those of the authors and do not necessarily represent those of their affiliated organizations, or those of the publisher, the editors and the reviewers. Any product that may be evaluated in this article, or claim that may be made by its manufacturer, is not guaranteed or endorsed by the publisher.
